# Modulation of Trichromatic Emission Centers in Organic–Inorganic Hybrids for Optoelectronic Applications

**DOI:** 10.1007/s40820-025-01965-0

**Published:** 2026-01-12

**Authors:** Weidong Cai, Chongyuan Li, Qiang Guo, Fuxiang Ji, Muyi Zhang, Yiqiang Zhan

**Affiliations:** 1 College of Future Information Technology, Fudan University, Shanghai, 200438 People’s Republic of China; 2 Institute of Computational Physics, Zurich University of Applied Sciences, Technikumstrasse 71, 8400 Winterthur, Switzerland; 3 Department of Physics, Chemistry, and Biology (IFM), Linköping University, 58183 Linköping, Sweden

**Keywords:** Trichromatic emission centers, Multicolor tuning, Chiral organic and inorganic halides, X-ray imaging, Latent fingerprints

## Abstract

Organic–inorganic metal halides (OIMHs) have emerged as highly promising novel multifunctional optoelectronic materials, owing to their easily adjustable properties from a variety of combinations of different components. But it is still difficult and rare to realize highly tunable multicolor luminescence within the same material. In this work, we successfully incorporated three adjustable emission centers in OIMHs to synthesize a novel OIMH (NEA)_2_MnBr_4_, with each emission center capable of emitting one of the primary colors—red, green, and blue. The green and red emissions originate from the tetrahedron and octahedron structures in the Mn-based frame, while the blue can be attributed to the contribution of organic components. Additionally, to achieve comparable emission intensity among the three primary colors, we enhanced the blue emission performance by optimizing the ratio of organic structure components and incorporating chirality in the OIMHs. The resulting high-quality films can be obtained by spin-coating method with a photoluminescence quantum yields of up to 96%. More interestingly, by the dual manipulation of excitation wavelength and temperature, the sample can be emitted at least seven distinct colors including a standard white luminescence at (0.33, 0.33), opening up promising prospects for multicolor luminescence applications such as high-end anti-counterfeiting technology, light-emitting diodes, X-ray imaging, latent fingerprints, humidity detection, and so on. Therefore, based on application scenarios and requirements, our research on this highly tunable luminescent OIMH material lays a solid foundation for further development of various functional properties of related materials.

**Supplementary Information:**

The online version contains supplementary material available at 10.1007/s40820-025-01965-0.

## Highlights

- A novel chiral (NEA)₂MnBr₄ was obtained by introducing three independently tunable primary-color emissive centers.
- Comparable emission intensities among the three primary colors were achieved by optimizing components and introducing chirality.
- By means of dual control of excitation photon energy and temperature, the sample can emit at least seven different colors, including standard white light emission at (0.33, 0.33).

**Supplementary Information:**

The online version contains supplementary material available at 10.1007/s40820-025-01965-0.

## Graphical abstract

**Figure:**
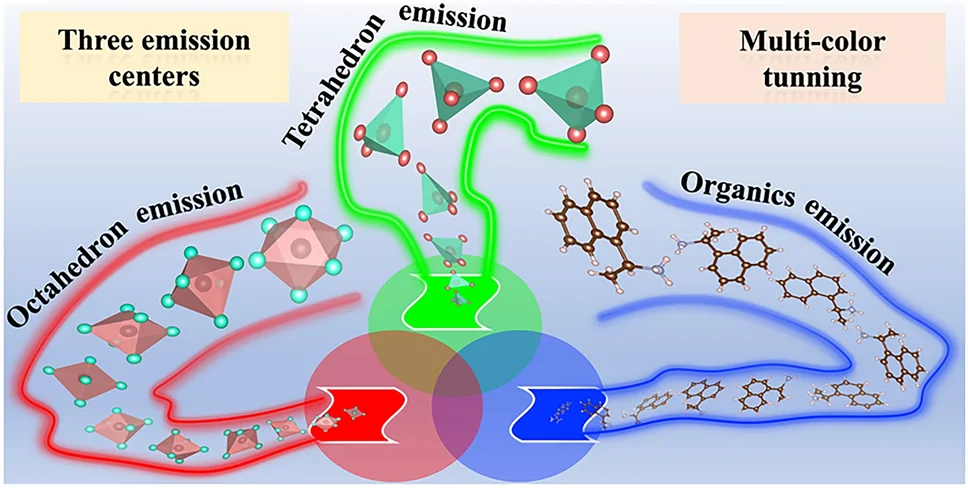

## Introduction

Organic–inorganic metal halides (OIMHs) have garnered increasing attention due to their multifunctional properties, which can be easily adjusted through the manipulation of organic and inorganic components. In order to develop novel functional photoelectric materials, various metal frameworks have been integrated into OIMH system to achieve a wide range of luminescent colors [1–8]. Among the counterparts, Mn-based OIMHs stand out for their potential to offer flexible tunability of emission colors and exceptional luminescence performance. This arises from the unique property of metal-centered *d-d* (^4^T_1_(G)–^6^A_1_) radiative transitions, enabling Mn-based OIMHs to exhibit strong green and red luminescence [9–16]. However, there is still a big limitation to produce independent and tunable trichromatic emission centers in Mn-based OIMHs. Traditional solutions such as metal doping or the combination of multiple phosphors have been attempted to obtain the desired colors [17–20]. Unfortunately, the lack of suitable metal dopants in the OIMH system and the complexities of the resulting molecular environment have posed significant challenges for further development and applications. Moreover, such conventional approaches typically yield fixed luminescent colors and increase the difficulty of adjustability.

Therefore, we are committed to developing Mn-based OIMHs with independent and adjustable trichromatic emission centers to achieve desired luminescence color according to specific practical requirements. In fact, the incorporation of organic coordination with blue emission can introduce directly independent blue luminescence centers in Mn-based OIMHs [21]. However, a notable drawback is the significantly weak intensity of the blue emission. Depending on the spatially separated property between organic coordination and inorganic metal frame, it is highly promising to overcome the disadvantage by effectively adjusting the relative ratio between organic and inorganic components and limiting the energy transfer to strengthen the contribution of blue emission.

Following this strategy, we introduced a highly blue-emissive organic ammonium salt, (Naphthyl)ethylamine (NEA), into Mn-based OIMHs as an individual blue emission center. Bromide manganese (MnBr_2_) is selected as the inorganic framework due to the ability of adjustable dual emission centers [22, 23]. On the one hand, we employed the spin-coating method to fabricate a high-quality film and adjust the ratio between organic and inorganic components to introduce more NEA component. On the other hand, we introduced chirality in organic coordination to reduce the energy transmission of blue light [24]. Benefiting from the synergistic effect of the above two approaches, the blue emission performance is improved significantly, the resulting three primary colors of luminescence were also successfully constructed with comparable luminescence intensities. Meanwhile, we can control the surface moisture of the (NEA)_2_MnBr_4_ film by heating to adjust the relative intensity between red and green emissions. Additionally, we found that varying the excitation wavelength is a comprehensive strategy for modulating the intensity relationship between three primary-color emissions due to significantly distinct excitation property of trichromatic emission centers. Thanks to this, we have achieved a wide array of desired emissive colors, including blue, purple, orange, pink, pure white, yellow, green, and more. This breakthrough enables the flexible production of desired colors within the same material, and this capability found in OIMHs opens the door to multifunctional applications in various luminescent fields. We applied it in high-end anti-counterfeiting, light-emitting diode (LED) emitters, X-ray imaging, and latent fingerprints (LFPs), and so on.

## Experimental Section

### Materials

(Naphthyl)ethylamine (NEA, 99%), hydrobromic acid (48 wt% in H_2_O, ≥ 99.99%), manganese (II) bromide (MnBr_2_, 99.9%), ethanol (C_2_H_6_O, 99.7%). All chemicals were commercially purchased from Sigma-Aldrich and used without further purification.

### Synthesis

Fabrication of (NEA)_2_MnBr_4_ single crystal. NEA (1 mmol), manganese (II) bromide (1 mmol), and hydrobromic acid (1 mmol) were dissolved in ethanol solution (30 mL), respectively. The two bottles of solution were heated at 50 °C on a heating platform and stirred for 30 min until they were completely dissolved, and then the two bottles of solution were mixed into a beaker and sealed and continued to heat at 50 °C and stir for 30 min until no precipitate appeared. Place the completely dissolved mixed solution in a dry fume hood, by slow evaporation of ethanol at room temperature for about one week, transparent crystals (NEA)_2_MnBr_4_ were obtained. The feed ratio of all organic–inorganic halide halides is about 60%-70%. When the air humidity is too high, supplement with heating at 40 to 60 °C. Finally, the material was subjected to a drying process and subsequently annealed in a furnace at around 60 °C for 1 h.

Fabrication of (NEA)_2_MnBr_4_ thin films. First, 100 mg of (NEA)_2_MnBr_4_ was dissolved in 350 µL ethanol solution. Then, the mixed solution was heated at 60 °C and stirred for 30 min. Spin-coating films were obtained by spin coating the solutions on glass substrates at around 3000 rpm, followed by annealing at 60 °C for 10 min.

**Scheme 1:**
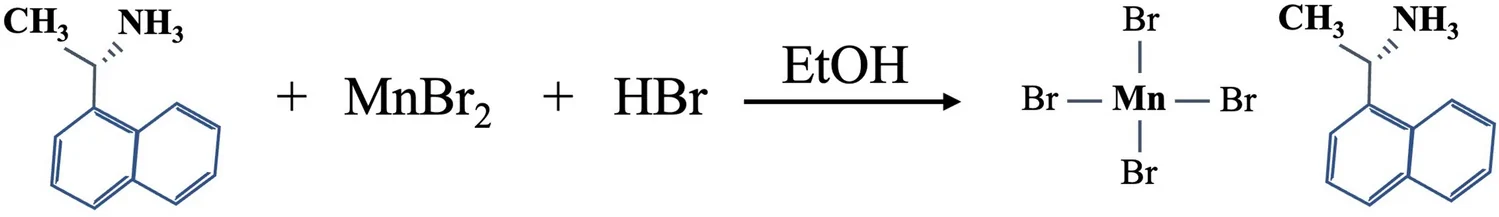
Chemical reaction progress for composite (NEA)_2_MnBr_4_

Fabrication of university logo films. 100 mg of the crystal of composite (NEA)_2_MnBr_4_ was dissolved in 350 µL ethanol solutions. Then, the mixed solutions were heated at 60 °C and stirred for 30 min. Logo film was obtained by spin coating the solution on indium tin oxide (ITO) substrates through shadow masks, followed by annealing at 60 °C for 10 min (Scheme 1).

### Characterization

X-ray diffraction (XRD) patterns of the products were recorded with X'Pert PRO X-ray diffractometer using Cu Kα1 irradiation (*λ* = 1.5406 Å). The radioluminescence was measured by the spectrometer (Edinburgh Instruments Ltd UC920 spectrometer) and the X-ray tube (M237, Newton Scientific) at a voltage of 30 kV. Energy-dispersive X-ray spectroscopy (EDS) analysis was performed using a LEO 1550 SEM operated at 18 kV accelerating voltage, with an Oxford Instruments X-Max 80 mm^2^ SDD detector. The film roughness was characterized using atomic force microscopy (AFM, Multimode 8, Digital Instruments). Steady-state photoluminescence spectra and photoluminescence quantum yield (PLQY) were recorded with Xenon light source as excitation and an Andor spectrometer (Shamrock sr-303i-B, coupled to a Newton EMCCD detector) as detection. Attenuated total reflectance (ATR-FTIR) mode was used for the FTIR spectroscopy characterizations. All spectra were recorded via a PIKE Miracle ATR accessory with a diamond prism in a Vertex 70 spectrometer (Bruker) using a DLaTGS detector at room temperature. The single-crystal X-ray diffraction data were collected at 298 K by using Cu Ka radiation on a Bruker D8 VENTURE single-crystal X-ray diffractometer (SCXRD) equipped with a kappa geometry goniometer. Data reductions and absorption corrections were performed with the APEX3 suite. Structures were solved by a direct method using the SHELXL-97 software package. The crystal structure was refined using full-matrix least squares based on F2 with all non-hydrogen atoms anisotropically defined. Hydrogen atoms were placed in calculated positions by means of the “riding” model. The details about data collection, structure refinement, and crystallography are summarized in Table S1.

## Results and Discussion

### Single-Crystal Structure and Luminescence Properties of OIMH Material

**Fig. 1:**
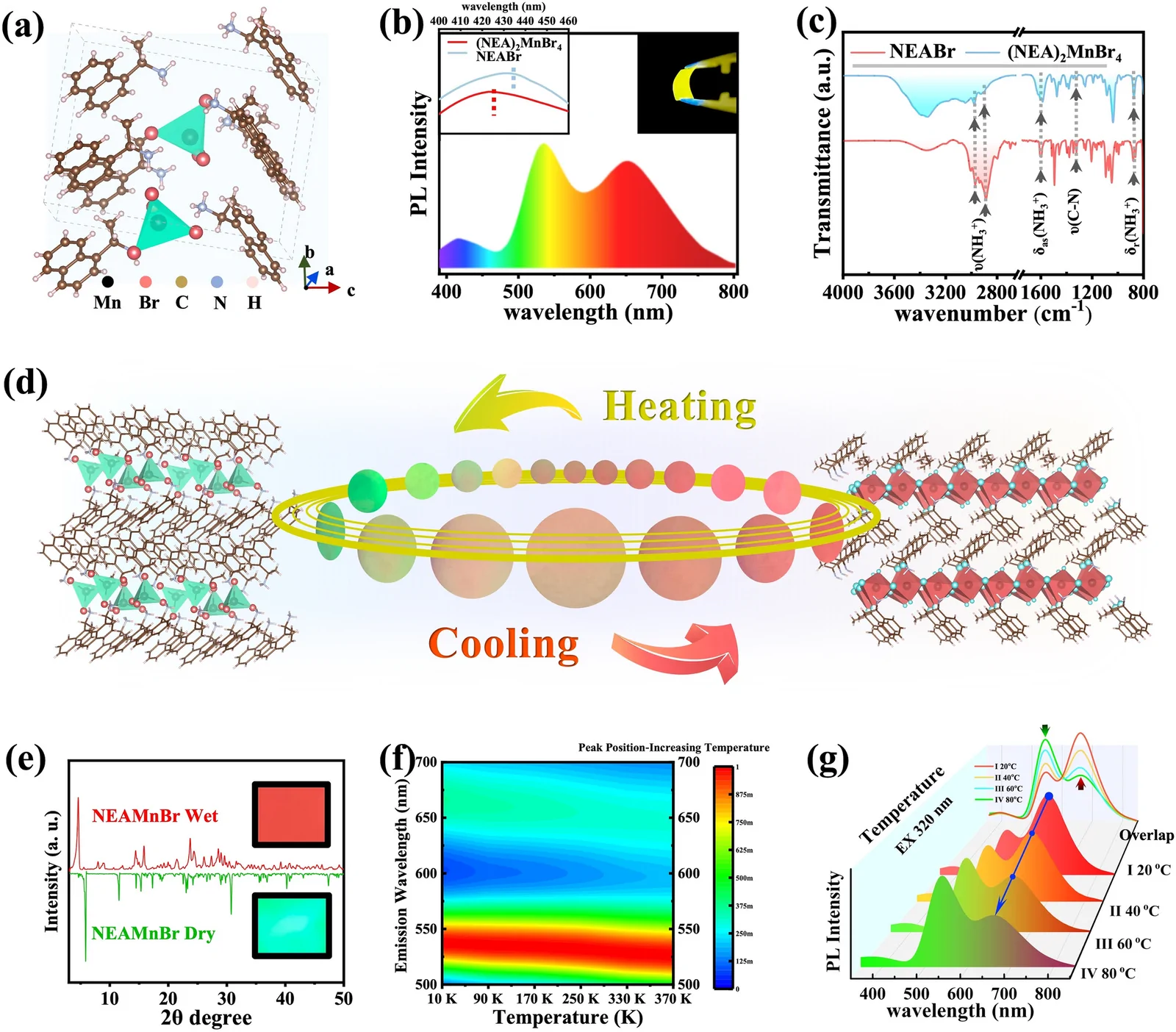
Structural and optical properties of (NEA)_2_MnBr_4_.** a** Crystalline structure of (NEA)_2_MnBr_4_. **b** Full PL spectrum of (NEA)_2_MnBr_4_ and corresponding optical photo of drop-casted (NEA)_2_MnBr_4_ film. The blue emission region of (NEA)_2_MnBr_4_ and NEABr is in the inserted figure. **c** Fourier transform infrared (FTIR) spectrum of (NEA)_2_MnBr_4_ film and precursor NEABr film. **d** Resulting color changes of samples by heating and cooling sample. **e** XRD patterns of (NEA)_2_MnBr_4_ under wet and dry environment. **f** PL spectra under vacuum conditions by varying the temperature. **g** PL spectra of (NEA)_2_MnBr_4_ film under different temperatures

We successfully obtained transparent OIMH single crystals of (Naphthyl)ethylamine manganese bromide ((NEA)_2_MnBr_4_) by a one-step method. This synthesis involved mixing manganese bromide (MnBr_2_) and (Naphthyl)ethylamine bromide (NEABr) in an alcohol solvent and allowing the mixture to sit at room temperature for one week. (The specific steps are shown in the synthesis section). A high crystallinity can be observed from the centimeter-scale single crystals (Fig. S1 inset). To ensure the high purity of our (NEA)_2_MnBr_4_ crystals, we performed X-ray diffraction (XRD) analysis, which closely matched the simulated single-crystal X-ray diffraction (SCXRD) patterns (Fig. S1). SCXRD revealed that OIMH possesses a zero-dimensional crystal structure with tetrahedral elements, as shown in Fig. 1a, also in line with Fig. S1 inset, where random orientations can be observed. The (NEA)_2_MnBr_4_ crystal consists of the orthorhombic phase and belongs to the P1 space group, with lattice parameters of *a* = 7.5935(2) Å, *b* = 11.6164(4) Å, and *c* = 15.4735(6) Å. The isolated tetrahedral [MnBr_4_]^2−^ units are surrounded by NEA organic molecules. The angles between two adjacent Br^−^ and Mn^2+^ ions (Br–Mn–Br) are approximately 110°, with bond distances between Br^−^ and Mn^2+^ ions measuring around 2.5 Å. This unique structure allows these crystal cells of Mn-based composites to maintain significant nanometer-scale molecular dimensions. The details about data collection, structure refinement, and crystallography are summarized in Table S1. Notably, MnBr_2_ and NEABr represent two separate and independent units due to the significant structural difference between the organic and inorganic components. The intrinsic quasi-ionic bonding effect and the substantial difference in structure between organic and inorganic units present an opportunity for the subsequent re-tuning of film compositions.

We can simply prepare (NEA)_2_MnBr_4_ thin films by dissolving the single crystals in ethanol and drop casting the solutions onto flexible polyethylene terephthalate (PET), followed by annealing at 60 °C. Our observations indicate that the drop-casted film exhibits three visible photoluminescence (PL) emission peaks, as shown in Fig. 1b: a blue emission at 425 nm with a full width at half maximum (FWHM) of 50 nm, a dominant green emission at 535 nm with a FWHM of 60 nm, and a red emission at 660 nm with a FWHM of 100 nm. The resulting flexible PET film displays a yellow emission, as shown in the inserted photo in Fig. 1b. The red or green emissions can be attributed to Mn-based structures [21–23]. The PL lifetime results reveal that the red emission has a significantly longer PL lifetime than the blue emission in (NEA)_2_MnBr_4_ (in Fig. S2), suggesting that the blue and red emissions in (NEA)_2_MnBr_4_ originate from distinct states. We synthesized precursor crystal NEABr and measured corresponding PL property to further verify the source of blue emission. As shown in Fig. S3, the dominant blue emission is centered around 435 nm with a FWHM of 25 nm, it can be visibly observed a significant red shift compared with (NEA)_2_MnBr_4_ (425 nm) according to the inserted figure in Fig. 1b, demonstrating that the significant difference of blue emission between crystal (NEA)_2_MnBr_4_ and the precursor NEABr. However, the similar excitation properties of NEABr and (NEA)_2_MnBr_4_ suggest their intrinsic connection. By comparing the photoluminescence excitation (PLE) of the blue emission between (NEA)_2_MnBr_4_ (emitting at 425 nm) and NEABr (emitting at 435 nm) in Fig. S4, almost identical PLE patterns can be observed, which differ from the PLE pattern of Mn-based emission characteristic in (NEA)_2_MnBr_4_. This comparison indicates that the contribution of the blue emission in (NEA)_2_MnBr_4_ comes from the emission center NEABr structure within (NEA)_2_MnBr_4_ rather than precursor NEABr. To further confirm the successful introduction of blue emission from the emission center NEABr, we compared the Fourier transform infrared (FTIR) spectra between the organic salt NEABr and the OIHM (NEA)_2_MnBr_4_ (in Fig. 1c). In the low-frequency region, nearly identical peak patterns were observed, especially the peaks at 1622, 1337, and 874 cm^−1^. These can be attributed to the antisymmetric bending mode δ_as_ (NH_3_^+^), the stretching mode υ(C-N), and the out-of-plane bending mode δ_r_ (NH_3_^+^) of NEABr, respectively. In the high-frequency region, the peak around 2800–3000 cm^−1^ can be attributed to the stretching mode υ(NH_3_^+^) of ammonium ions. The similarity in the FTIR peak patterns between (NEA)_2_MnBr_4_ and NEABr confirms the presence of the emission center NEABr structure in our Mn-based composite, which is also consistent with the results of PL, PLE, and PL lifetime.

Having identified the sources of blue, red, and green emission colors, it becomes beneficial to understand and apply the chromism phenomenon stemming from the susceptibility of OIMH (NEA)_2_MnBr_4_, which exhibits moisture-dependent PL [23]. By carefully controlling the heating temperature, we were able to manipulate the surface moisture and observed the resulting color changes, as shown in Fig. 1d. When cool down the sample, the sample color becomes red, and as the temperature increases, the color changes from red to green. Behind the color change, the structure transfer process has happened between octahedrons and tetrahedrons according to numerous reports [12, 23]. We also observed different XRD patterns of wet sample with red color and dry sample with green color in Fig. 1e. The dry sample with green emission exhibits characteristic peaks at 5.8° and 11.5° in low degree region, while the wet sample with red emission displays markedly different characteristic peaks at 4.5°, 8.0°, 8.8°, 10.6°, and 12.2°. These obvious disparities in XRD patterns clearly indicate that the inner structure changes behind the process of color change. Notably, temperature variation under humid conditions involves changes in both temperature and humidity. We then measured PL under vacuum conditions by varying the temperature, and we observed that the relative intensity between the high-energy and the low-energy peak did not change significantly over a wide temperature range from 10 to 370 K (Fig. 1f), indicating that humidity is indeed the cause of the PL difference. To further quantify the color changes in Fig. 1d, we performed the corresponding PL measurements, as shown in Fig. 1g. When the heating temperature was set at 20 °C in stage I, the PL exhibited a dominant red emission peak at 660 nm with a FWHM of 135 nm, and a green emission peak at 535 nm with a FWHM of 55 nm. As the heating temperature increased from stage I at 20 °C to stage IV at 80 °C, the PL intensity of the green emission grew progressively stronger, while the red emission gradually weakened until the pattern ultimately shifted into a dominant green emission peak and a weaker red emission peak. The corresponding Commission Internationale de l'éclairage (CIE) coordinates clearly exhibit color transfer process as shown in Fig. S5. The color of the sample film transitioned from an orange emission region (I) with CIE coordinates of (0.47, 0.46) through yellow emission regions to a light green emission region (IV) with CIE coordinates of (0.39, 0.52). However, the color span is still not wide enough in CIE coordinates spectra through the manipulation of heating temperature, which is difficult to reach the requirements of multicolor tuning for broad range of application scenarios. The challenge mainly lies in the relatively weaker blue emission compared to the red and green emissions.

### Optimization of the Organic Components and the Introduction of Chirality Yield Three Primary-Color Peaks with Comparable Intensities

**Fig. 2:**
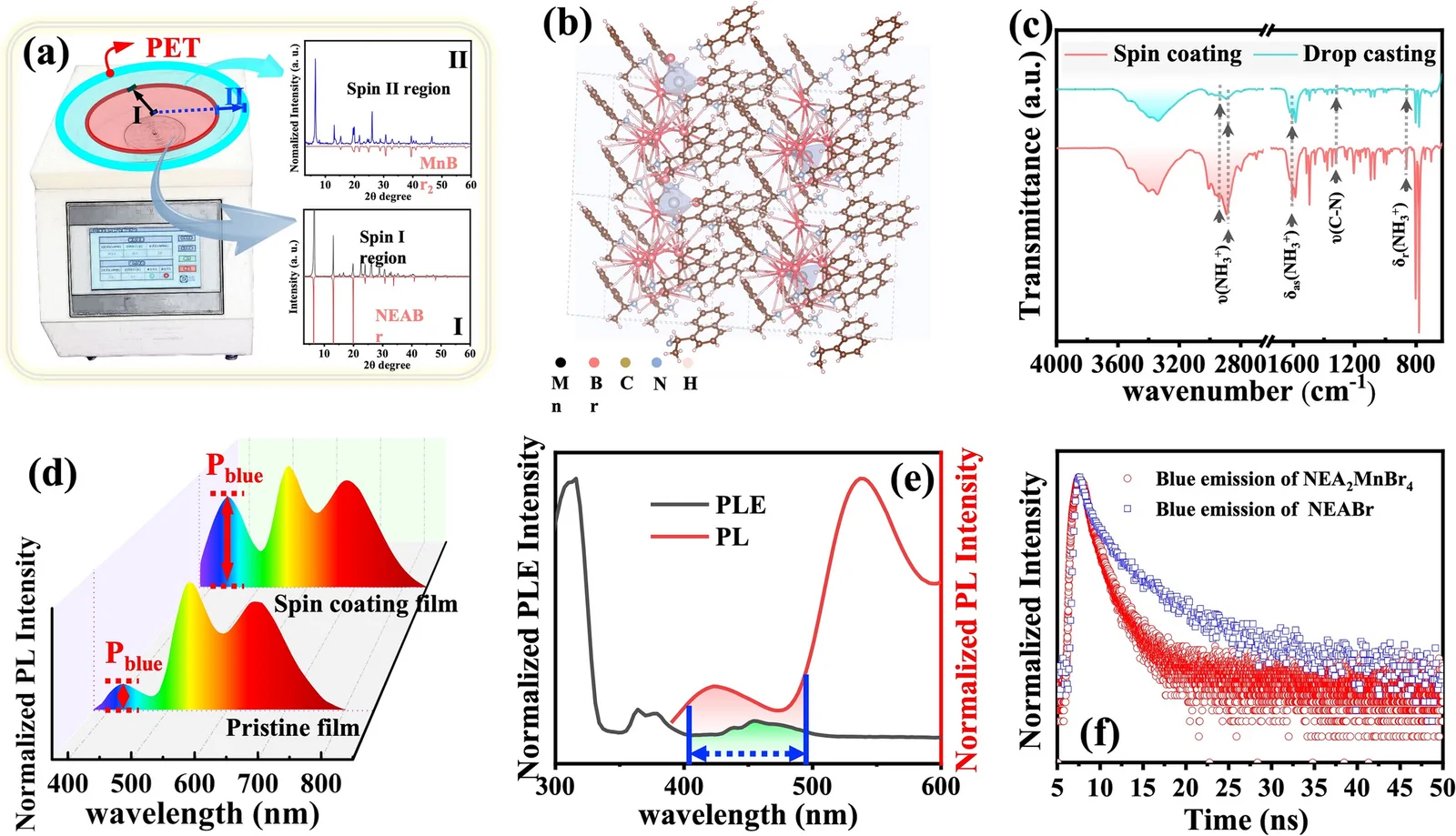
Re-optimization and characterization of the components in OIMH, along with evidence of energy transfer. **a** Schematic diagram of spin coater with big PET film onside, the right picture is the XRD of the corresponding area on the PET. **b** Schematic diagram of the crystal structure of (NEA)_2_MnBr_4_ film with denser organic components. **c** FTIR absorption spectra of (NEA)_2_MnBr_4_ spin-coated and drop-casted films. **d** PL spectra of pristine film and spin-coating film. **e** Overlap area of blue emission and green excitation. **f** PL lifetime of (NEA)_2_MnBr_4_ and precursor NEABr. (Color figure online)

Interestingly, based on the unique structure of OIMH, it provides intrinsic weak quasi-ionic bonding and spatial separation properties between organic ligands and inorganic metal framework. This offers an opportunity to tune the relative proportions of each component within the OIMHs. Given such difference, the spin coating is an effective method to leverage centrifugal force to separate the distinct phases. As expected, we observed a marked solubility distinction between the organic and inorganic precursors. MnBr_2_ exhibited a solubility of 20 mg mL^−1^, whereas NEABr displayed a substantially higher solubility of 305 mg mL^−1^, as depicted in Fig. S6. The considerable difference in structure and solubility may lead to distinct distributions of different components on the substrate during the spin-coating process. We also conducted separate spin-coating tests with NEABr and MnBr_2_ precursors on ITO substrates. We found that during the spin-coating process, the MnBr_2_ easily precipitates from the solvent onto the substrate surface in Fig. S7a, while the more soluble NEABr precursor remains in solution to form an even film in Fig. S7b. This illustrates that MnBr_2_ is indeed easy to precipitate during the spin-coating process. Under high-speed centrifugation, the more soluble components, such as the organic component (NEABr), tend to remain uniformly on the substrate in solution form. In contrast, due to the combined effects of its low solubility and the high volatility of the ethanol solvent, the metal halide (MnBr₂) can rapidly precipitate and be expelled from the substrate. Therefore, many particles can usually be observed on the inner wall of the spin coater in Fig. S7c. To observe this phenomenon more prominently, we chose a bigger terephthalate (PET) substrate (Diameter: 10 cm) to spin coat our (NEA)_2_MnBr_4_ solution as depicted in the schematic diagram of Fig. 2a. We divided the substrate into inner region I and outer region II and compared the XRD patterns of the corresponding two regions after spin coating. Due to the simple component and peak information of precursors of MnBr_2_ and NEABr, it can be clearly observed that film in region I contains a mass of NEABr by common feature peaks such as 6.2°, 13.2°, 19.8°, and 23.8°, while the external film in region II shows significantly different XRD patterns from the inside film in region I, which exhibits similar XRD patterns with MnBr_2_ by common feature peaks such as 15.2°, 19.6°, 21.4°, 28.9°, 30.8°, and 33°. It demonstrates external film in region II contains a large amount of MnBr_2_ due to low solubility and rapid crystallization process, which is thrown out to the periphery as particle states. Conversely, the precursor NEABr with high solubility is more retained as solution on substrate. The result is also consistent with energy-dispersive X-ray spectroscopy (EDS) result in Fig. S8, the pristine crystal exhibited a 1/4 ratio of Mn to Br, while the film in the external region II shows higher ratio of Mn and Br (> 1/3) than that in the inside I region (< 1/6).

After the spin-coating process, followed by annealing and crystallization, the increased organic component (NEABr) and less transfer barriers from polyhedral structure within the structure led to the formation of a denser NEABr bonding effect in the film sample, as illustrated in the structural schematic diagram in Fig. 2b. This tighter molecular environment induced a significantly stronger bonding energy between the organic NEA and the halogen Br, which can also be observed in FTIR measurements (Fig. 2c). The spin-coated film exhibited a stronger signal intensity for the NEABr bonding energy in the antisymmetric bending mode δ_as_(NH^3+^), the stretching mode υ(C-N), the out-of-plane bending mode δ_r_(NH^3+^), and the stretching mode υ(NH^3+^) when compared to the drop-cast film. It demonstrates denser organic components are successfully introduced. Another visible evidence is the significantly improved blue emission of spin-coated films compared to pristine drop-coated films in Fig. 2d, illustrating the contribution of the increased organic structure NEABr within structure.

**Fig. 3:**
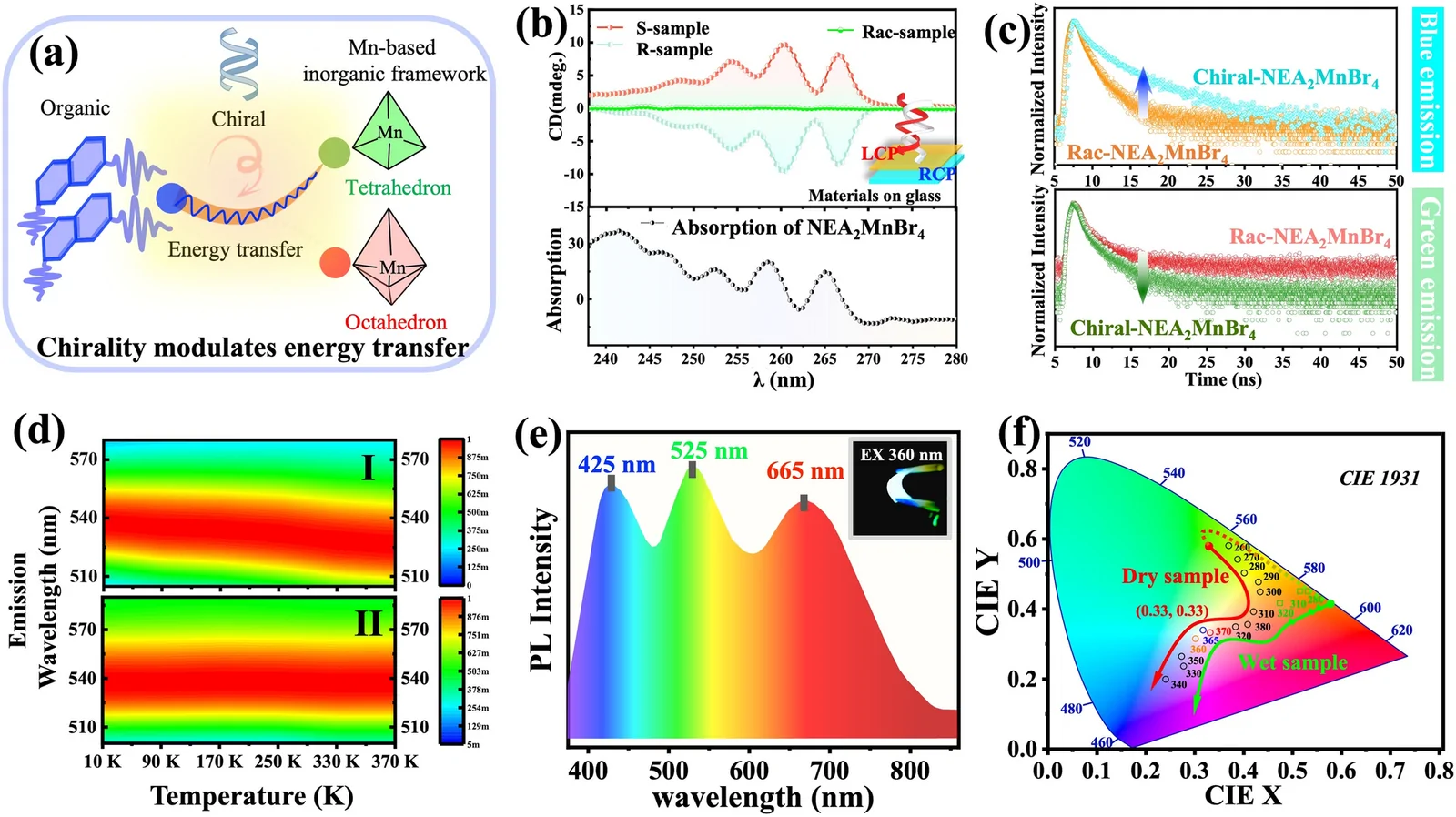
Introduction of chirality and adjustment of three primary colors. **a** Schematic diagram of chiral-controlled energy transfer. **b** Transmission CD spectra of R-(NEA)_2_MnBr_4_, S-(NEA)_2_MnBr_4_, and RAC-(NEA)_2_MnBr_4_ films and the corresponding absorption positions. **c** Variation trend of blue and green PL lifetimes before and after the introduction of chirality. **d** Under variable temperature conditions, the green light emission of RAC-(NEA)_2_MnBr_4_ shows a blue shift (I), and the green light emission of chiral-(NEA)_2_MnBr_4_ remains stable at 540 nm (II). **e** PL spectrum and corresponding optical photo of chiral (NEA)_2_MnBr_4_ film with three excitation peaks of equal intensity. **f** CIE coordinates of dry and wet (NEA)_2_MnBr_4_ film under different excitation wavelength. The red curve is the color gamut distribution of the dry sample, and the green curve is the color gamut distribution of the wet sample. (Color figure online)

Meanwhile, energy transfer between different emission centers may also affect the intensity of blue emission. As shown in Fig. 2e, green emission has significant excitation signals in the 400–500 nm range, which aligns with the blue emission wavelength in Fig. 2e. This suggests that the blue emission from organic ligands can cause energy transfer to the green emission of tetrahedral structures. By comparing the PL lifetimes of (NEA)_2_MnBr_4_ and single-crystalline NEABr (in Fig. 2f), we found that single-crystalline NEABr exhibits a longer lifetime. This is primarily due to the significant overlap between the emission band of the organic component and the absorption band of Mn^2^⁺, enabling energy transfer from the organic component to the Mn^2^⁺ green emission center. Chen et al. found that the chiral matching between the donor and acceptor has a significant impact on the energy transfer process. When the chirality is mismatched, the energy transfer is often significantly suppressed [24]. The incorporation of chirality enables polarized blue emission by reducing the matching degree of dipole–dipole interactions, altering the local electric field distribution, and introducing spin-selective effects. Therefore, as illustrated in the schematic diagram in Fig. 3a, we introduced chirality into the organic ligands to suppress the energy transfer of blue emission and lead to a more balanced trichromatic emission, thereby achieving better full-color emission regulation.

We introduced chiral NEA into the Mn-based inorganic framework and measured the circular dichroism (CD) spectra of the resulting composite films (Fig. 3b). For the S/R–(NEA)_2_MnBr_4_, distinct CD signals were observed in the range of 238 to 280 nm, indicating that the composite films prepared with chiral NEA possess chirality. The peaks in the CD spectra of the S/R–(NEA)_2_MnBr_4_ exhibit opposite signs, corresponding to S- and R-polarization, respectively. In contrast, the racemic (NEA)_2_MnBr_4_ shows a flat CD signal, indicating the absence of chirality. The absorption spectra are also shown in Fig. 3b, demonstrating that the chiral absorption region lies within the optical transition range of our materials. Our experimental results confirm that chirality has been successfully incorporated into sample films. We were unable to reliably measure CPL in thin films, as the signal was indistinguishable from slight PL fluctuations under continuous excitation. The coexistence of weak CPL and strong CD can generally be attributed to strong ground-state chirality, but weak or decoupled excited-/emissive-state chirality [25]. We further investigated the changes of our sample in blue and green PL lifetimes before and after the introduction of chirality. We found that, after introducing chirality, the blue emission lifetime increased significantly, while the green emission lifetime decreased notably (Fig. 3c). This is because the energy transfer from the blue emission to the red and green emission centers typically occurs via Förster resonance energy transfer (FRET) or Dexter electron exchange mechanisms. The introduction of chiral organic ligands disrupts the alignment between the dipole moments of the blue donor and those of the red/green acceptors, thereby reducing the efficiency of Förster energy transfer. At the same time, the incorporation of chiral structures also disrupts orbital coupling, suppressing the Dexter energy transfer process [26, 27]. As a result, blue energy is less likely to be transferred to Mn^2^⁺ centers, leading to prolonged excited-state lifetimes for the blue emission. With fewer excitons received by the Mn^2^⁺ structure, the green emission lifetime is consequently shortened. We also measured the PL spectra of the materials before and after the introduction of chirality under variable temperature conditions (Fig. 3d). During the heating process from 10 to 370 K, the green emission exhibited a blue shift from 540 to 525 nm (I), while the green emission of chiral-(NEA)_2_MnBr_4_ remained stable at 540 nm (II). Typically, as the temperature increases, the energy transfer mechanisms (Dexter or Förster) from the organic blue emitter to Mn^2^⁺ are enhanced. This leads to increased emission from Mn^2^⁺ centers with lower crystal field strength located at the blue end of the spectrum, resulting in a more pronounced blue shift [28]. The introduction of chirality blocks the energy transfer from blue emission to Mn^2^⁺, thereby suppressing the temperature-induced blue shift and further improving the blue light intensity (Fig. S9).

### Dual Regulation of Excitation Photon Energy and Temperature Enables Multicolor Tuning and High-performance Applications in Diverse Scenarios

With the enhancement of organic component and the inhibition of energy transfer, we finally achieved three peaks of comparable intensity, enabling us to produce pure white emission light (0.33, 0.33) after spinning coat (NEA)_2_MnBr_4_ material on a flexible PET film, as shown in Fig. 3e. In the meantime, the adjustability of temperature-dependent emission color can be further improved under an excitation wavelength of 330 nm, as demonstrated in Fig. S10. When a high heating temperatures (80 °C) is placed, the sample can emit strong green emission light and weaker red emission light. Interestingly, when the temperature was lowered to 20 °C, the luminescence phenomenon of the two peaks was reversed. The red emission peak began to rise until it became dominant, while the green emission peak gradually weakened. During this process, the corresponding color span is visually represented by the CIE coordinates in Fig. S11. The color of the film transitioned from a green emission region (I) with CIE coordinates of (0.35, 0.41) through various light yellow emission regions to the final pink emission region (VI) with CIE coordinates of (0.37, 0.34). With the enhanced blue emission component, the resulting color gamut distribution could move closer to the central region compared to the CIE coordinates of the red curve with weak blue emission by drop casting. However, the color gamut span is still small, and practical colors like pure white light are still missing, which will limit the development of wider applications. Depending on distinct excitation property between different emission centers (Fig. S4), the excitation wavelength can be used as a variable to adjust the emission color. Therefore, we introduced an excitation wavelength-dependent PL strategy to tune the emission intensity of all peaks, further enhancing the multicolor tunability of our thin film samples.

Firstly, we use the control variable method to maintain constant low humidity by encapsulating the sample film after high-temperature heating. The color span of the dry sample can be observed when the excitation wavelength is switched from 260 to 380 nm. The corresponding CIE coordinates and the color gamut span are depicted in the red curve in Fig. 3f. Specifically, a variety of distinct colors can be obtained, including visible green emission with CIE coordinates of (0.37, 0.58), yellow emission with CIE coordinates of (0.40, 0.50), orange emission with CIE coordinates of (0.43, 0.45), pink emission with CIE coordinates of (0.38, 0.35), pure white emission with CIE coordinates of (0.33, 0.33), purple emission with CIE coordinates of (0.28, 0.24), and light blue emission with CIE coordinates of (0.24, 0.20). We could obtain at least seven distinct colors on a single material. Then, we can also maintain another constant high humidity by encapsulating the sample film at a lower temperature (20 °C). Similarly, we can observe the color span of the wet sample by varying excitation wavelength. Compared with the dry sample, the color gamut distribution of the wet sample is similar near the blue and white color gamut, but the green color gamut is replaced by the red color gamut like (0.53, 0.45) and (0.52, 0.45) due to a dominated contribution from red emission of octahedron structure, as shown in green curve in Fig. 3f. Therefore, for both dry and wet samples, adjusting different excitation wavelengths can induce a Y-shaped distribution in the color gamut, which further enriches the application possibilities of materials according to different practical application scenarios. Notably, as shown in Fig. 1d, the red-to-green emission transition induced by heating or cooling is reversible, which enables us to conveniently tune the Y-shaped distribution region according to practical needs, thereby enhancing the material’s capability for color gamut modulation.

Beyond the enhanced blue light and a wider color gamut distribution, the quality of the film was also significantly improved through the spin-coating process, as depicted in Fig. S12. Traditional drop-casting technology, commonly used in the OIMH system, typically results in a rough surface crystallinity, as illustrated in Fig. S12a. This is due to the difficulty in controlling the film thickness, the uneven evaporation rate of the solvent, and the coffee ring phenomenon that is easily produced under the rapid crystallization effect, which leads to poor film quality. In contrast, the spin-coated film displayed a very smooth surface morphology, as seen in Fig. S12b. This is because the spin-coating method can provide the necessary centrifugal force through high-speed rotation, helping adjust the film thickness and allowing the solution to spread quickly and uniformly. To further rule out negative effects of the spin-coating process on normal-sized films, such as phase separation or abnormal crystallization, we collected atomic force microscope (AFM) and EDS data from both the central and edge regions of the films and constructed histograms to evaluate the process stability (Fig. S13). We found that the surface roughness as well as the Mn/Br ratios in the two regions is very similar. This demonstrates that the spin-coating process does not induce significant crystallization differences or phase separation in small-area films; such pronounced compositional variations only emerge when the film size is more than an order of magnitude larger. Thanks to the good film quality and improved luminescence from the three emissive centers, we were able to achieve high photoluminescence quantum yields (PLQY) of up to 96%, as indicated in Fig. S14. Therefore, we can apply this film to fields such as LED devices or imaging that require strong luminous intensity and high film quality.

**Fig. 4:**
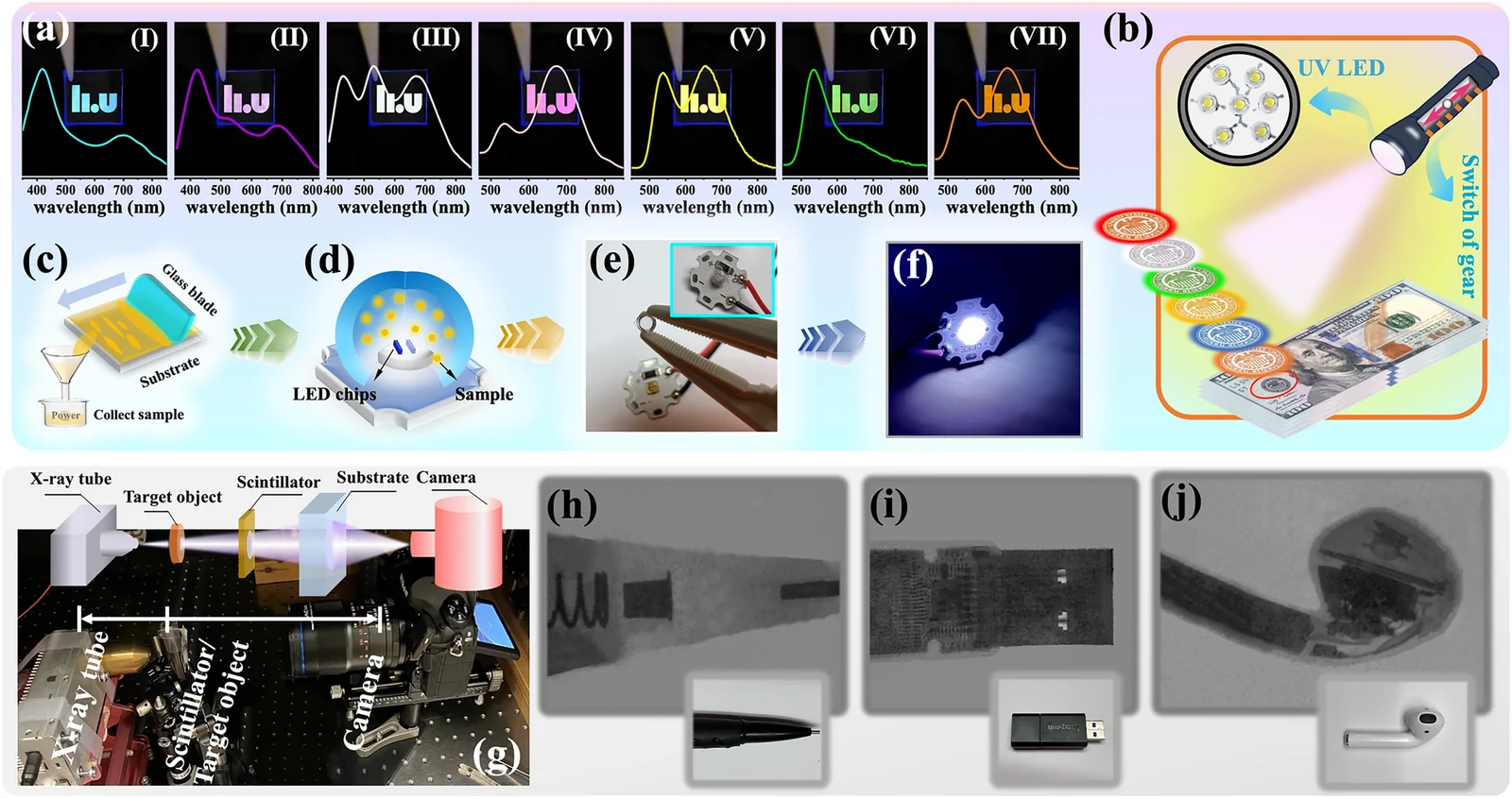
Applications of our OIMH in high-end anti-counterfeiting, LED emitter devices and X-ray imaging. **a** I-VII University logo exhibits significant color differences under different excitation wavelengths. **b** Schematic diagram of banknotes anti-counterfeiting technology by printing our transparent and flexible material onside. **c** Process of collecting sample powder. **d**, **e** Schematic diagram of the LED device with the powder of (NEA)_2_MnBr_4_ film inside of LED glass and the corresponding optical picture. **f** Photograph of the working device operated at 10 mA. **g** Schematic of the as-designed indirect X-ray imaging system. **h-j** Bright field images of a pen, a USB disk, and an AirPods earphone and their X-ray images based on the (NEA)_2_MnBr_4_ screen. (Color figure online)

**Fig. 5:**
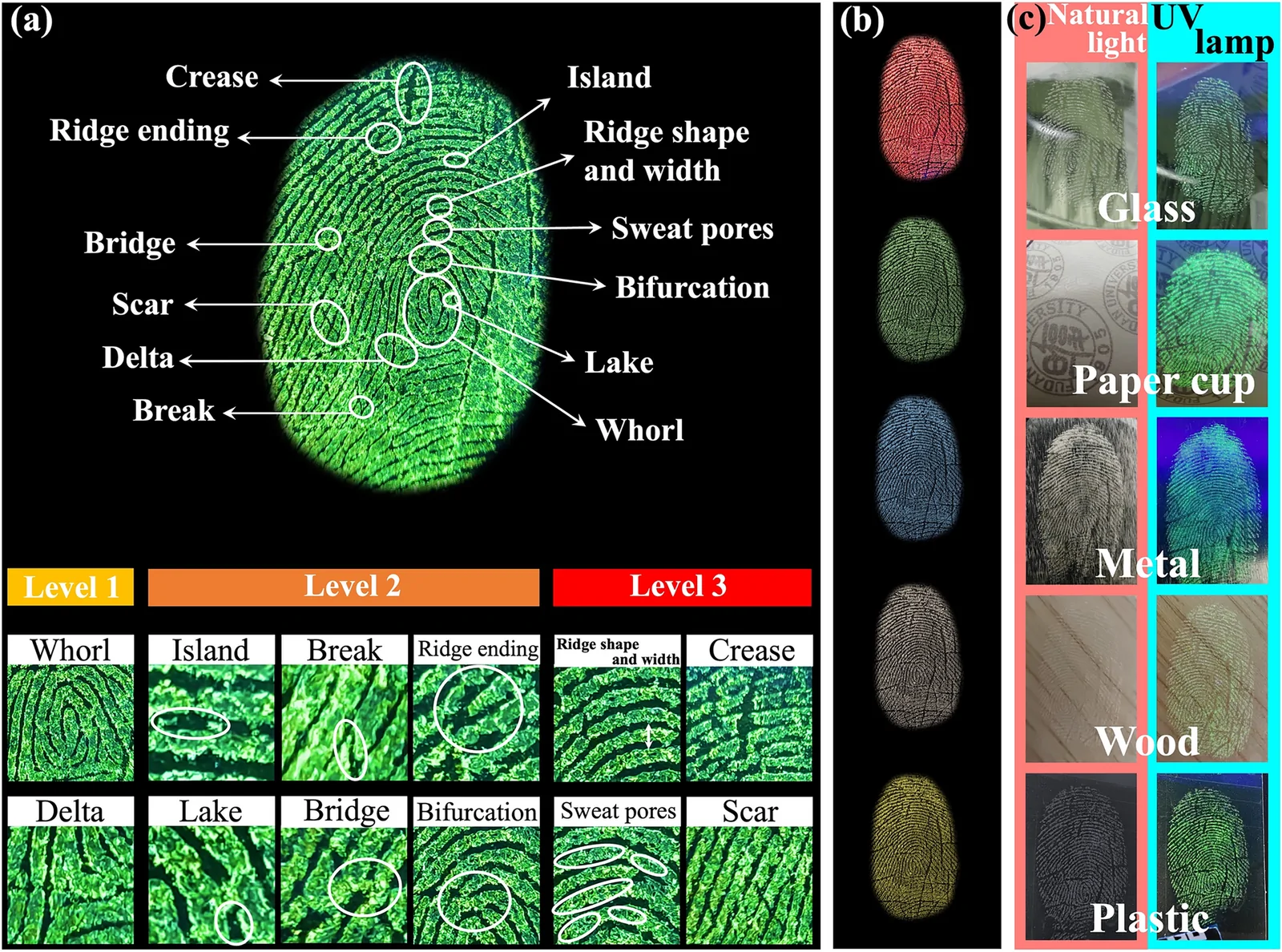
Application of our materials in LFP. **a** Magnified view of detail of power (NEA)_2_MnBr_4_-stained LFP on a glass slide. **b** LFP images of (NEA)_2_MnBr_4_ powder taken on glass under switching the UV lamp excitation wavelength. **c** Bright field and fluorescence images of latent fingerprints (LFPs) can be developed by coating our material powder on various surfaces such as glass, paper, metal, wood, and plastic

Our material not only exhibits excellent optical properties but also demonstrates long-term stability after simple encapsulation. As illustrated in the inset photo in Fig. S15b, our sample is sandwiched between two layers of PET, with the edges sealed by AB glue, which can effectively block air and moisture. Even after two years of storage, the structure and luminescent properties remain well-preserved according to XRD and PL results as depicted in Fig. S15, demonstrating the durability of the encapsulated material. After achieving stability and durability, we can obtain at least seven different emission colors on this material by just a simple UV lamp with tunable excitation wavelength, showing great potential for applications in high-end anti-counterfeiting technology [29–35]. To demonstrate the practical application of our concept in anti-counterfeiting, we dissolved the (NEA)_2_MnBr_4_ material in an alcohol solution and spin-coated it onto a PET or ITO substrate. By using different shadow masks, we created various patterns, such as the university logo, as displayed in Fig. S16a. When exposed to UV light of different excitation wavelengths, the logo exhibited distinctly different colors, as shown in Fig. 4a I–VII: a light blue logo at 340 nm, a purple logo at 330 nm, a white logo at 370 nm, a pink logo at 320 nm, a yellow logo at 280 nm, a green logo at 260 nm, and an orange logo at 300 nm. If this transparent and flexible material is printed on banknotes, antiques or luxury goods labels, as shown in the schematic diagram in Fig. 5b, simply illuminating it with UV lamps of different wavelengths to excite specific luminescence would elevate the anti-counterfeiting level significantly. Consequently, after satisfying the criteria of uniqueness, stability, detectability, and feasibility required for anti-counterfeiting materials, we believe that our new material holds great potential for application in high-end anti-counterfeiting technology.

Among various types of emission colors, white light is particularly versatile and can often be used for many application scenarios, such as lighting and displays. In this work, we obtained a quite pure white light emission with CIE coordinates of (0.33, 0.33) and applied it to the light-emitting diodes (LEDs) emitter [36]. We firstly scraped the samples on the substrate after spin coating and collected the powder as shown in schematic diagram in Fig. 4c, which was then attached it inside the protective glass of LED chips (as depicted in schematic diagram of Fig. 4d and optical pictures of Fig. 4e). When the LED is powered, the pure white light can be emitted, as shown in Figs. S16b and 4f. The external quantum efficiency (EQE) of the pristine bare-chip LED was 3.9%, whereas the EQE of the phosphor-coated conversion device decreased to 2.0%, due to additional scattering, reabsorption, and non-radiative processes introduced by the phosphor layer. The current–voltage (I–V) characteristics (Fig. S17b) exhibit typical diode-like rectifying behavior: The leakage current is negligible at low bias, but once the applied voltage exceeds the threshold voltage (V_th_) of approximately 3.0 V, the current density increases sharply, consistent with the wide-bandgap nature of the UV emitter. Spectral measurements (Fig. S17a) indicate that the phosphor-converted LED achieves a broad emission wavelength range from 380 to 780 nm, covering the entire visible spectrum. The corresponding correlated color temperature (CCT) is 5542 K, within the neutral white light range. Moreover, the device exhibits a high color rendering index (CRI) of 92, demonstrating excellent color fidelity under illumination. Detailed CRI values (Fig. S17c) for the reference colors (R1–R8) range from 86 to 96, further confirming the superior color quality. This makes it ideal for solid-state lighting applications requiring broad spectral coverage and high color fidelity. In fact, more emission light colors can be excited by using small LED bulbs with different excitation wavelengths or those that can automatically switch wavelengths, allowing for further development of LED applications according to specific requirements.

Meanwhile, we also found that strong green emission can be achieved under 260 nm excitation. Combined with the high-quality films produced by spin coating, this offers promising application prospects for (NEA)_2_MnBr_4_ in X-ray imaging [37–40]. This is because X-ray imaging typically demands high-quality films and strong green emission, as the scintillator layer is required to attenuate sufficient high-energy photons meanwhile the photodetectors like charge-coupled devices (CCD) and complementary metal-oxide semiconductor (CMOS) demonstrate high quantum efficiencies in the 500–600 wavelength range. Radioluminescence (RL) measurements on the sample film confirmed a strong RL signal with bright green emission, as shown in Fig. S18. To demonstrate the potential of our OIMHs film, we employed our homemade X-ray imaging optical system to evaluate the X-ray imaging performance of the (NEA)_2_MnBr_4_ film as depicted in the schematic diagram in Fig. 4g. We ensured the film's durability by dipping it in an organic protective solution (95% methylbenzene and 5% polyethylene), allowing it to maintain its luminescence properties in the open air for over one year. We selected a pen, a USB flash drive, and AirPods as target objects with increasing levels of identification difficulty. The spring components inside the pen, the numerous tiny silver bracket components of the USB drive, and the intricate internal details of the AirPods can be captured in fine detail under X-ray irradiation (Fig. 4h–j). By optimizing the thin film spin-coating preparation process and using a standard line pair card for benchmarking (in Fig. S19a), it was measured that the spatial resolution of X-ray imaging can reach 14–16-line pairs per millimeter (lp mm^−1^) (in Fig. S19b). The non-toxicity, low synthesis cost, and exceptionally high-resolution place it at a leading position among both hybrid and inorganic systems.

Due to the uniqueness of fingerprints, human fingerprint recognition has become a powerful tool at crime scenes. In latent fingerprints (LFPs) detection technologies, various development approaches have emerged, as the results are often influenced by the properties of the substrate surface, operational procedures, and environmental conditions. Among these, the powder dusting method has become the most widely used technique in forensic departments due to its environmental friendliness and low labor intensity. Typically, fingerprint features are classified into three levels. Level 1 refers to macroscopic features such as whorls and delta, which are insufficient for identifying local fingerprint details. Level 2 describes finer ridge features, including ridge endings, bifurcations, bridges, and so on. Level 3 consists of microscopic features such as the shape, scar, and location of sweat pores, providing quantitative data for personal identification [41]. Traditional powder dusting methods often suffer from poor color contrast and limited detail resolution. In our organic–inorganic hybrid materials, the functionalized organic components can interact with lipids, proteins, or amino acid residues in fingerprints via hydrogen bonding or π–π stacking interactions, facilitating high-resolution LFP detection. As shown in the enlarged fingerprint image on glass in Fig. 5a, detailed fingerprint features such as ridge crossings and bifurcations, endings, scars, and sweat pores can be clearly observed. The inorganic component provides a stable framework and excellent tunable optical properties, allowing our scintillators to exhibit distinct luminescence under UV light (Fig. 5b). This effectively minimizes background interference, and combined with the wide applicability of the material on a variety of surfaces (Fig. 5c), enables it to have high recognition efficiency on surfaces with various patterns, colors, or different textures. We also extracted the grayscale values (G) of the selected green line area in Fig. S20 using ImageJ software. The resolution of LFP showed a high contrast between the papillary ridges and the groove/basal surface, as evidenced by the difference in fluorescence intensity. Therefore, our hybrid material has the potential to be promoted as a novel material for powder dusting methods.

In addition, the humidity-dependent PL characteristics of this material can also be used for humidity detection as illustrated in Fig. S21. Through the P value of the ratio of the red emission peak intensity P_r_ to the green emission peak intensity P_g_, we can obtain a linear relationship between the P value and the humidity level. Therefore, this material has a wide range of adjustment capabilities and application development prospects.

## Conclusions

In our research, we achieved coexisting red and green emissions by integrating a Mn-based metal framework with both octahedral and tetrahedral structures. Then, NEA with strong blue emission is incorporated into Mn-based framework, so that we can successfully obtained (NEA)_2_MnBr_4_ crystal with three primary emission colors. For further enhancing the blue emission component, we firstly employed the spin-coating method to tune ratio relationship between organic and inorganic components to obtain films. Then, we introduced chiral NEA to restrain the energy transfer of blue emission. As a result, we achieved three primary colors with comparable emission intensity. Moreover, we could fine-tune the relative intensity between the three emissions by excitation wavelength and temperature. We can obtain at least seven distinct emission colors, demonstrating the potential for high-end anti-counterfeiting applications, LED emitter, and LFPs. We also harnessed this material as a scintillator for X-ray imaging due to its strong green emission and RL signal. The material exhibited clear X-ray imaging and maintained good longevity with encapsulation, showcasing its potential for long-term use in high-resolution X-ray imaging. The multifunctional applications underscore the immense potential of our Mn-based OIMHs, which can be further custom-designed and harnessed in optoelectronics field.

## Supplementary Information

Below is the link to the electronic supplementary material.

Supplementary file1 (DOCX 2487 KB)
