# Supplementary material for: Modulation of Trichromatic Emission Centers in Organic–Inorganic Hybrids for Optoelectronic Applications
Source: Nanomicro Lett. 2026 Jan 12;18:140. doi: 10.1007/s40820-025-01965-0 (PMC12791107; doi:10.1007/s40820-025-01965-0)

Supporting Information for

**Modulation of** **T****richromatic Emission Centers in Organic–Inorganic Hybrids for Optoelectronic Applications**

Weidong Cai^1^, Chongyuan Li^1^, Qiang Guo^1^, Fuxiang Ji^2^, Muyi Zhang^3^, Yiqiang Zhan^1^*

^1^College of Future Information Technology, Fudan University, Shanghai 200438, P. R. China

^2^Institute of Computational Physics, Zurich University of Applied Sciences, Technikumstrasse 71, Winterthur 8400, Switzerland

^3^Department of Physics, Chemistry, and Biology (IFM), Linköping University, Linköping SE-58183, Sweden

*Corresponding author. E-mail: [yqzhan@fudan.edu.cn](mailto:yqzhan@fudan.edu.cn) (Yiqiang Zhan)

**Supplementary Figures and Tables**


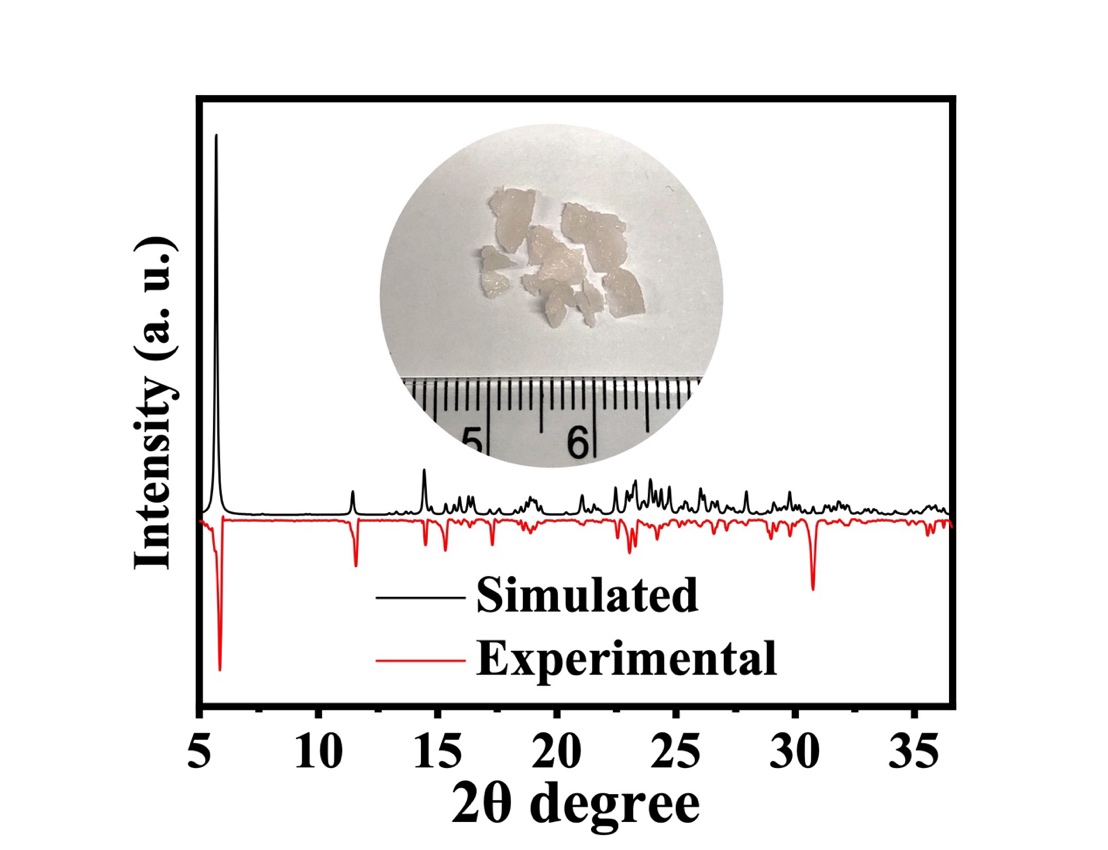


**Fig. S1** Experimental and simulated XRD patterns with inserted optical photo of (NEA)_2_MnBr_4_ crystal

**Fig. S2** PL decay curves of the (NEA)_2_MnBr_4_ at the emission wavelengths of 425 nm and 660 nm


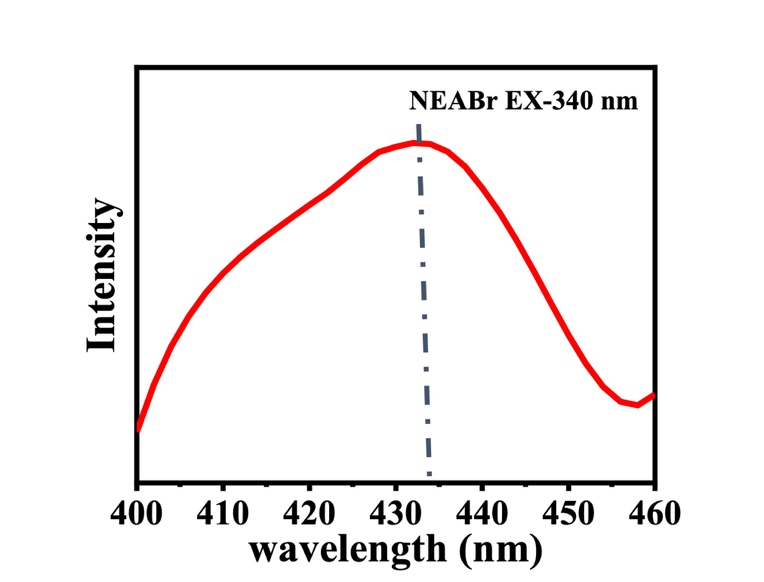


**Fig. S3** PL spectra of precursor NEABr under excitation wavelength of 340 nm

**Fig. S4** Photoluminescence excitation (PLE) pattern of precursor NEABr film at emission peak 435 nm and (NEA)_2_MnBr_4_ film at emission peak 425 nm and 660 nm


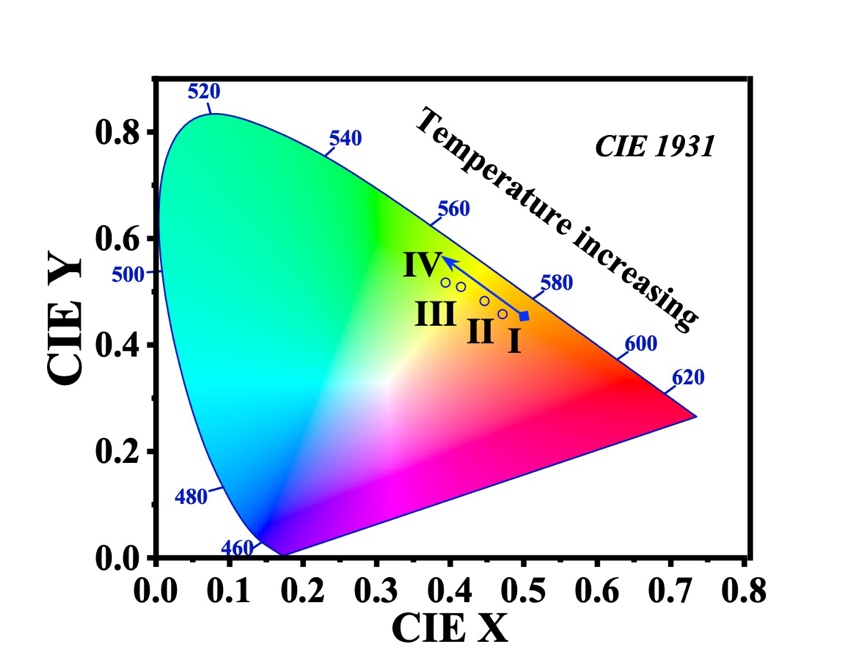


**Fig. S5** CIE coordinates of (NEA)_2_MnBr_4_ film under different temperatures


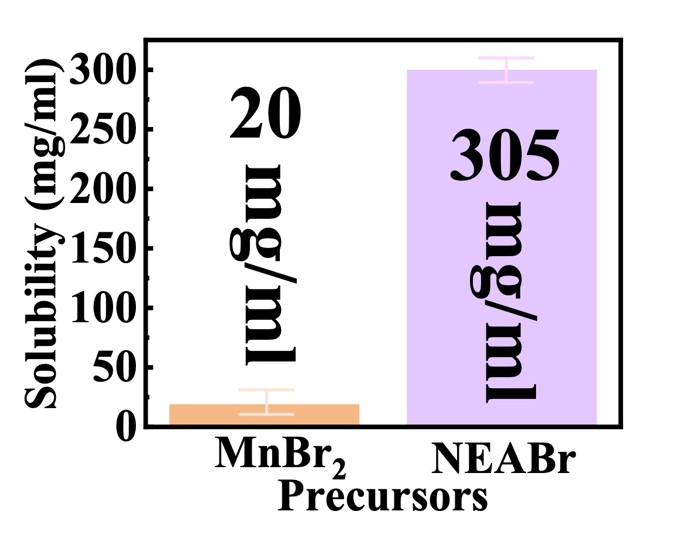


**Fig. S6** Solubility bar graph of precursors NEABr and MnBr_2_


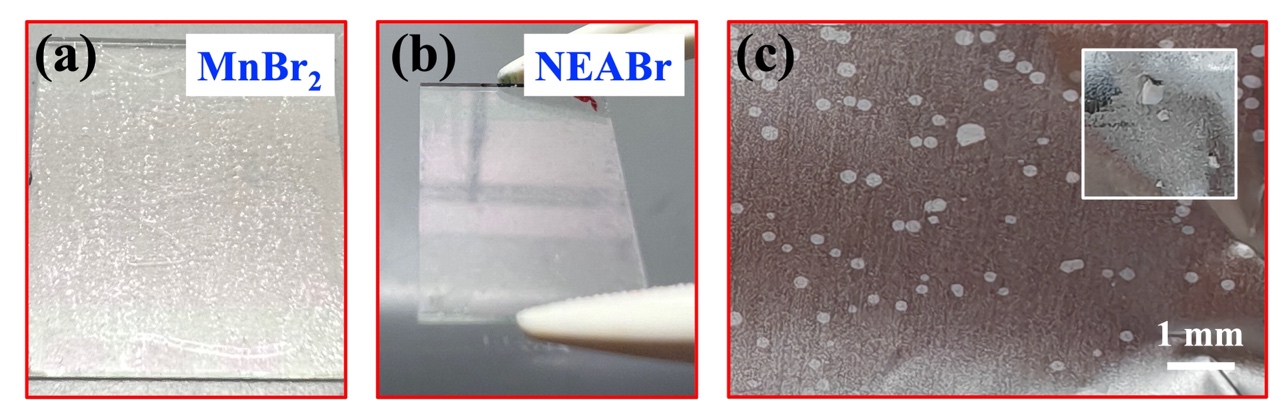


**Fig. S7** The spin-coated film of precursors (**a**) MnBr_2_ and (**b**) NEABr (**c**) MnBr_2_ particles on the tin foil inside the spin coater


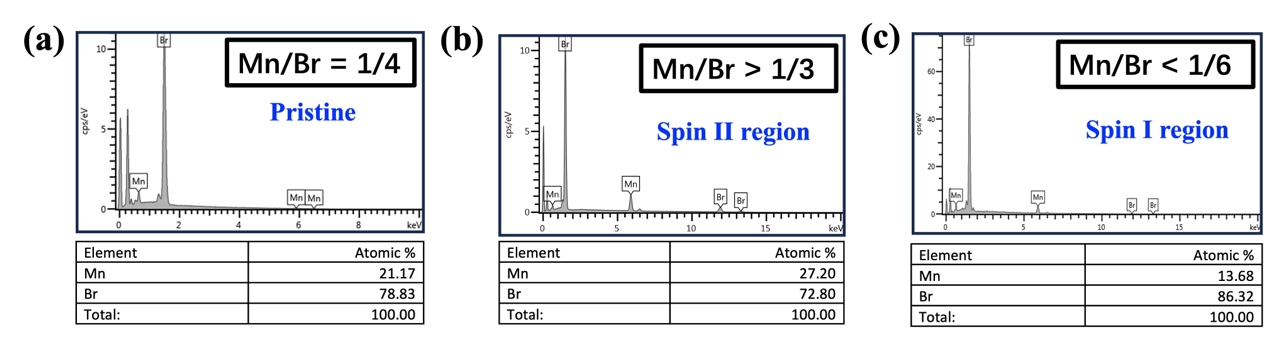


**Fig. S8** Energy dispersive X-ray spectroscopy (EDS) results of (**a**) pristine crystal (NEA)_2_MnBr_4_, (**b**) spin-coated films in region II and (**c**) spin-coated films in region I


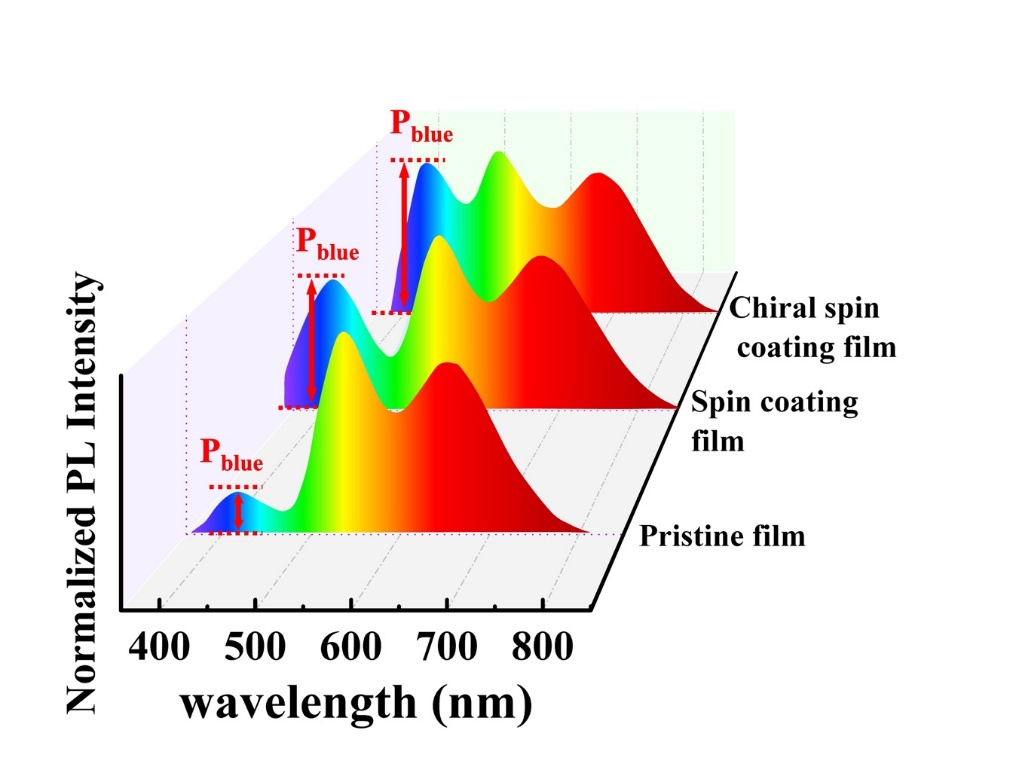


**Fig. S9** PL spectra of pristine film, spin coating film and chiral spin coating film
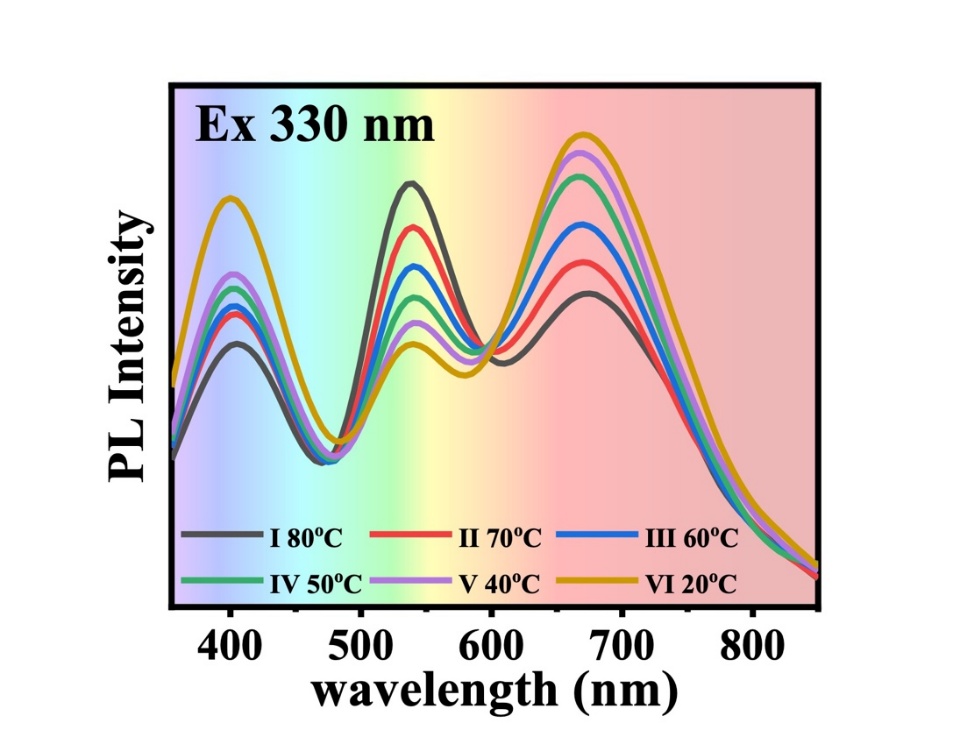


**Fig. S10** PL spectrum of (NEA)_2_MnBr_4_ film under different temperatures


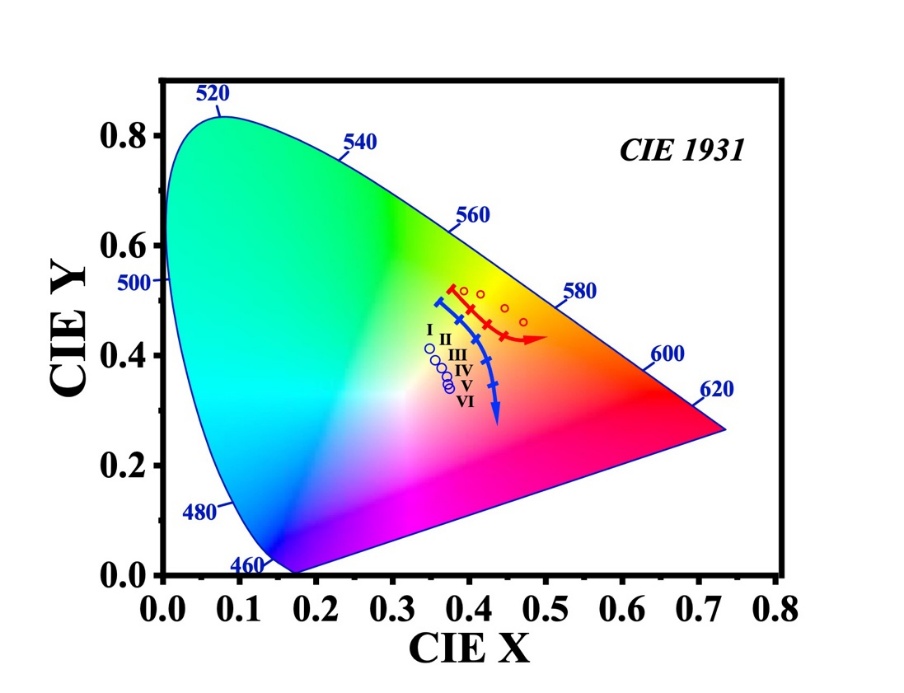


**Fig. S11** Corresponding CIE coordinates of spin coating film and drop casting film under different temperatures


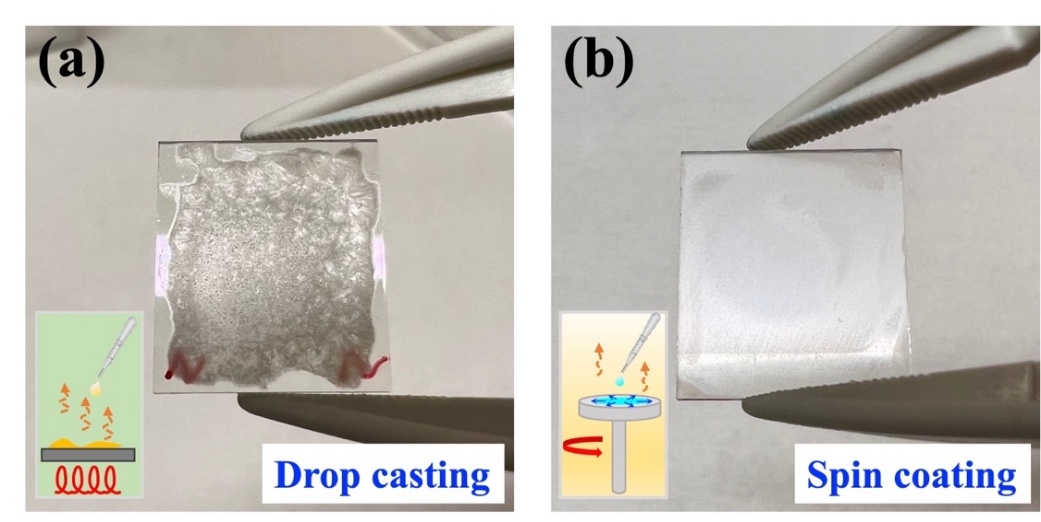


**Fig. S12** The (NEA)_2_MnBr_4_ film is obtained by (**a**) the drop casting method and (**b**) the spin coating method


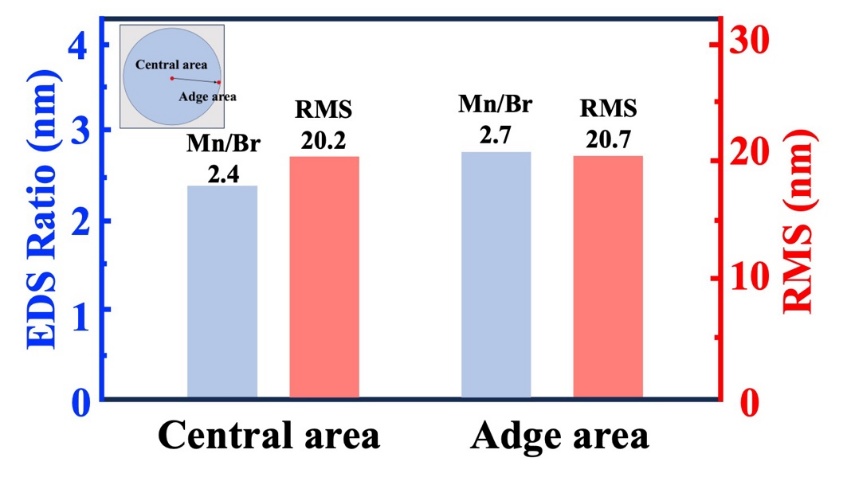


**Fig. S13** Statistical histogram of film component ratio (EDS) and roughness (AFM) in the center and edge areas of the spin-coated film


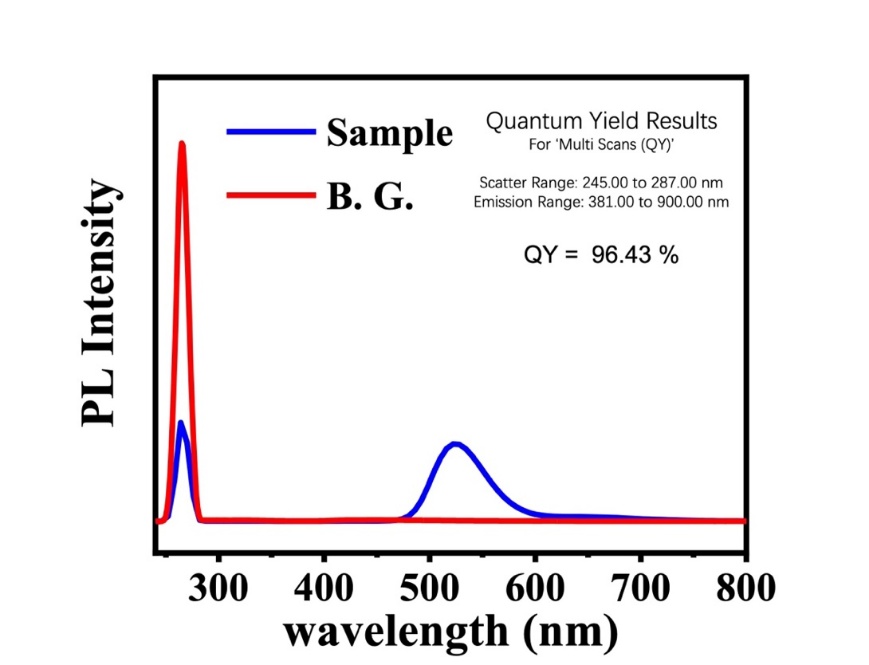


**Fig. S14** The PLQY of (NEA)_2_MnBr_4_ film


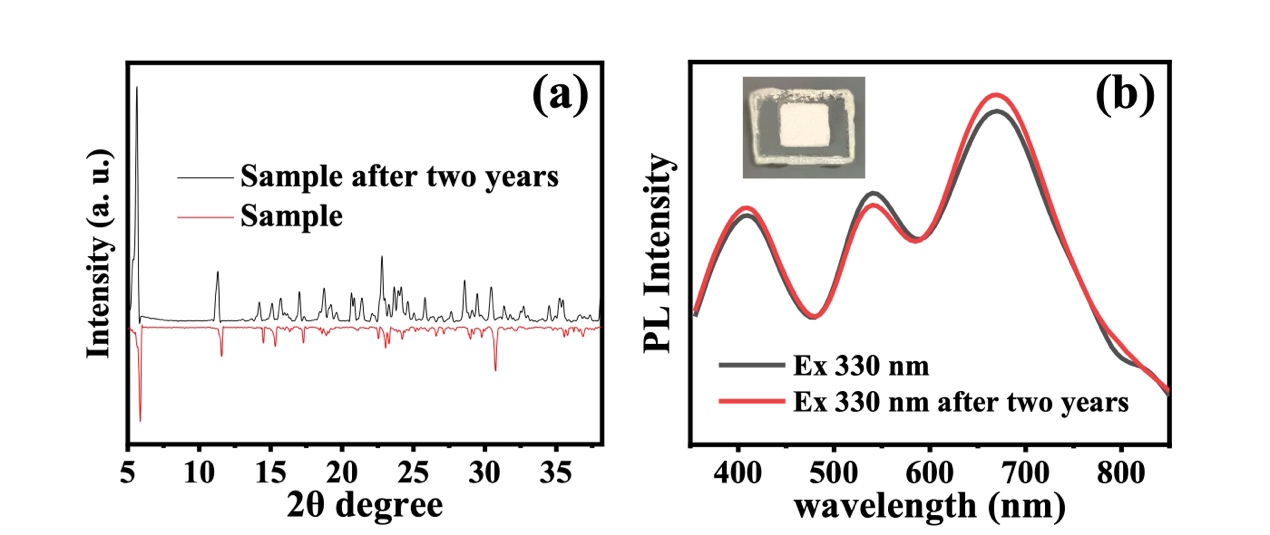


**Fig. S15** XRD and PL Stability measurements of (NEA)_2_MnBr_4_ after sample with encapsulation is placed in air after two years


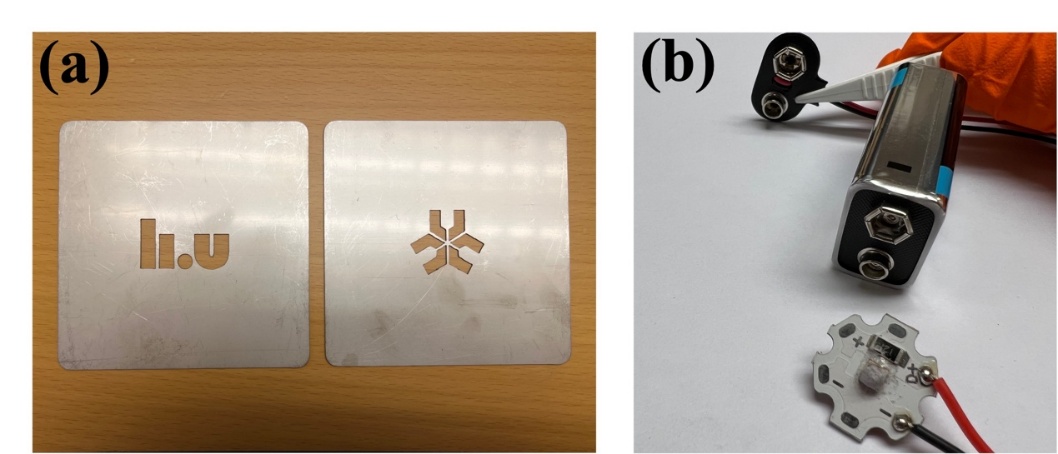


**Fig. S16** (**a**) Templates for spray-painting the university logo. (**b**) We collect powder after spin coating on substrate, then cover them inside the protective glass of LED chips to light up the LED


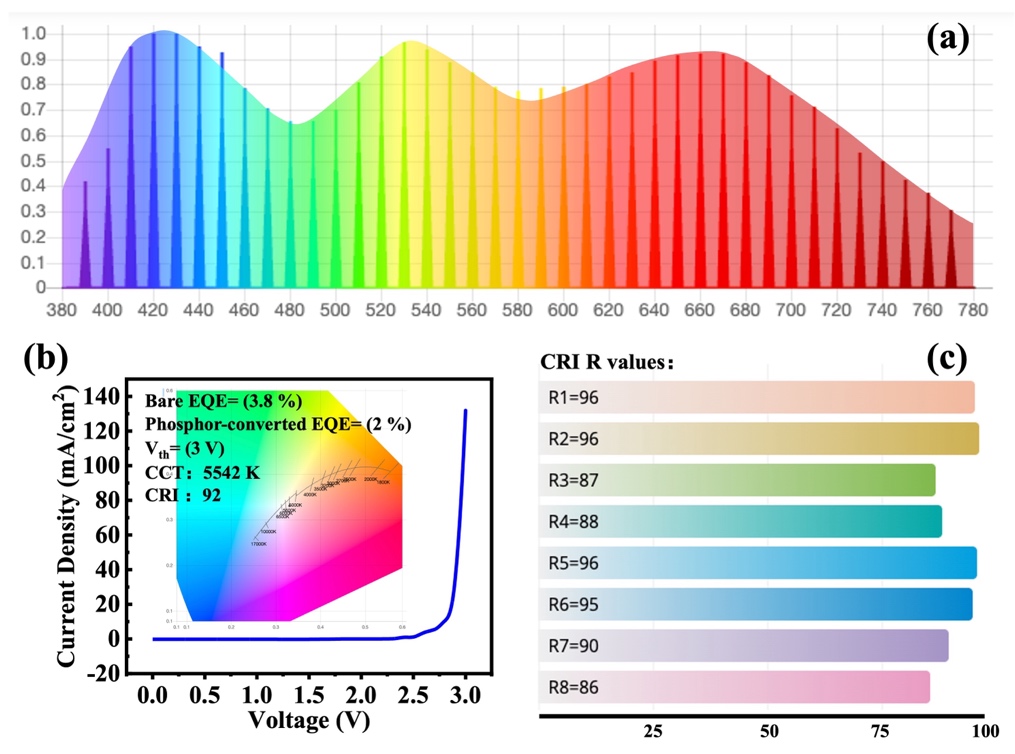


**Fig. S17** (**a**) Emission spectrum of white LED. (**b**) J–V curve of the fabricated photoluminescent excitation device, the inserted image is the CCT (Correlated Color Temperature) coordinates. (**c**) R1–R8 values of the device’s white-light emission with CRI90 (Color Rendering Index) in the wavelength range of 380–780 nm

**Fig. S18** Radioluminescence (RL) spectrum of (NEA)_2_MnBr_4_ film


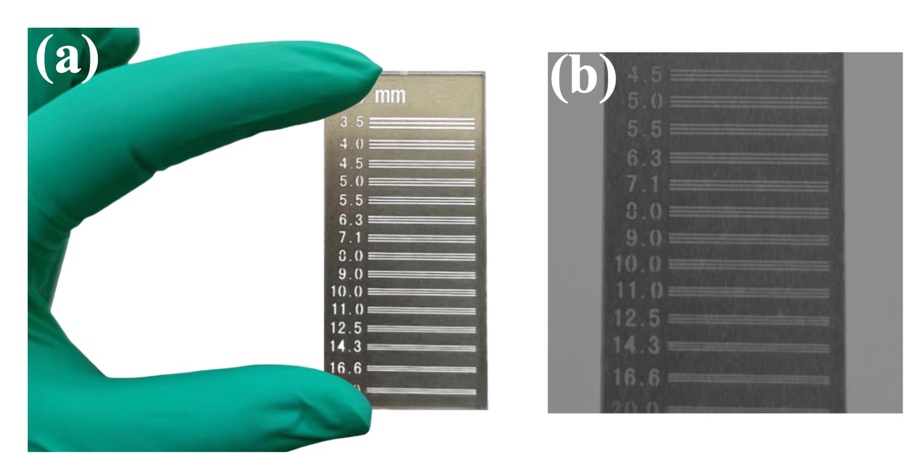


**Fig. S19** X-ray image of the standard X-ray resolution pattern plate with scintillator and its bright-field image


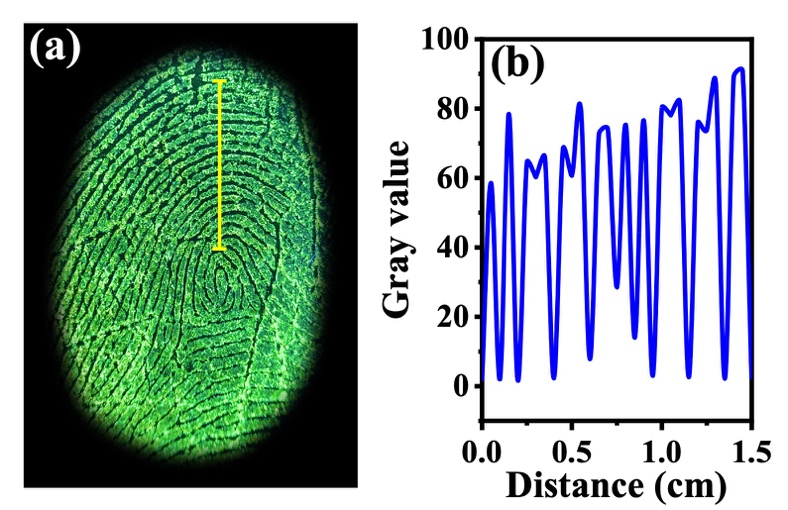


**Fig. S20** The LFP grayscale distribution map was obtained along the direction indicated by the yellow straight line of the stained LFP


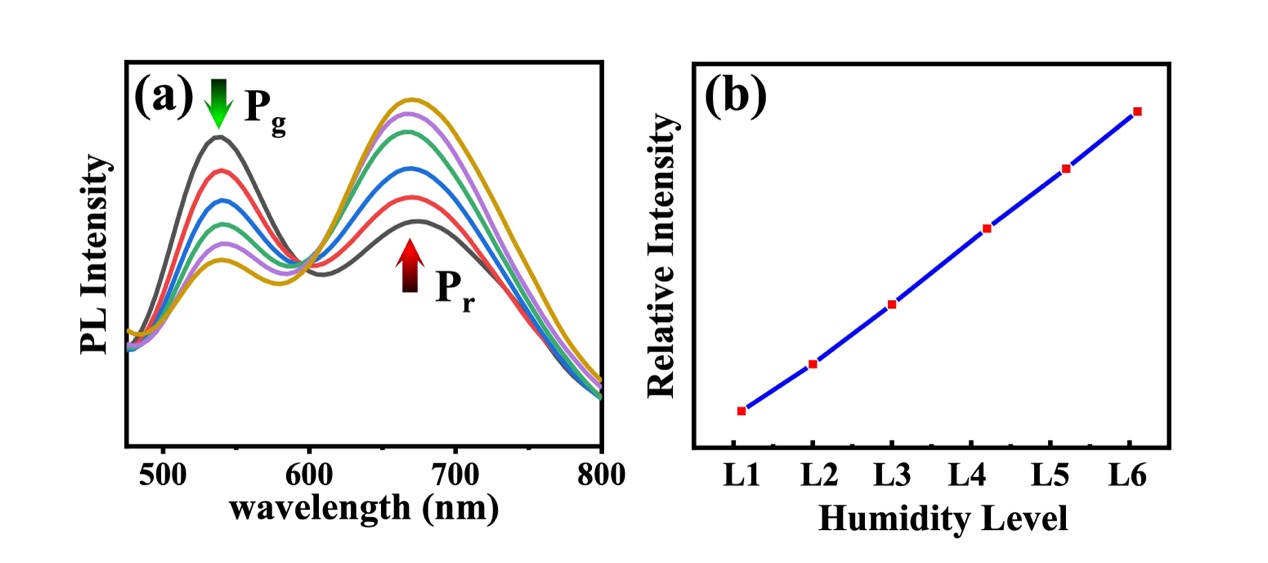


**Fig. S21** (**a**) Humidity-dependent PL spectra. (**b**) The linear relationship between the P value (P=P_r_/P_g_) and the humidity level

**Table S1** Crystallographic data of (NEA)_2_MnBr_4_

| Empirical formula | C_24_H_28_Br_4_MnN_2_ |
| --- | --- |
| Formula weight | 719.06 |
| Temperature/K | 150.0 |
| Crystal system | triclinic |
| Space group | P_1_ |
| a/Å | 7.5935(3) |
| b/Å | 11.6164(4) |
| c/Å | 15.4735(6) |
| α/° | 90.849(2) |
| β/° | 90.779(2) |
| γ/° | 96.480(2) |
| Volume/Å^3^ | 1355.87(9) |
| Z | 2 |
| ρ_calc_ g/cm^3^ | 1.761 |
| μ/mm^-1^ | 6.395 |
| F(000) | 702.0 |
| Data collection range/° | 4.368 to 52.862 |
| Index ranges | -9 ≤ h ≤ 9, -14 ≤ k ≤ 14, -19 ≤ l ≤ 19 |
| Goodness-of-fit on F^2^ | 0.941 |
| Final R indexes [I>=2σ (I)] | R_1_ = 0.0329, wR_2_ = 0.0666 |
| Final R indexes [all data] | R_1_ = 0.0443, wR_2_ = 0.0714 |
| CCDC no. | 2300440 |


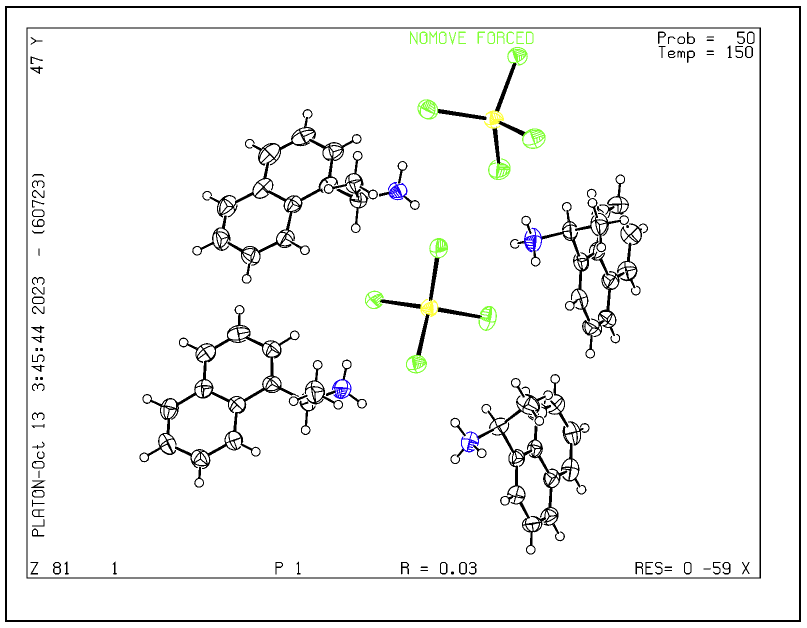

Supplement: Supplementary file 1 — Supplementary file1 (DOCX 2487 KB) [file 40820_2025_1965_MOESM1_ESM.docx]
